# Palatoradicular Groove–Associated Periodontic–Endodontic Lesion: A Multidisciplinary Case Report Utilising Minimal Access Papilla-Sparing Regenerative Technique (MAPSRT) and Dual-Flap Approach

**DOI:** 10.1155/crid/5073663

**Published:** 2025-11-05

**Authors:** Amelia Hemmati, Stephanie Chan, Mehdi Valizadeh, Robert Childs, Leticia Algarves Miranda, Paul Abbott, Pradeep Koppolu

**Affiliations:** UWA Dental School, The University of Western Australia, Perth, Australia

**Keywords:** case report, palatoradicular groove, periodontal surgery, regenerative therapy

## Abstract

This case report describes the management of a palatoradicular groove on a lateral incisor tooth (#12) in a nonperiodontitis patient. Despite the commencement of endodontic treatment, the tooth showed no signs of resolution and was referred to the Oral Health Centre of Western Australia for specialist management. Tooth 12 presented with a periodontal probing depth of 7 mm at the mid-palatal site with suppuration. Nonsurgical periodontal therapy adjunct with the application of 0.5% chlorhexidine gel was completed, followed by the completion of endodontic treatment one week prior to regenerative therapy. A minimal access papilla-sparing regenerative technique (MAPSRT), a novel, site-specific modification, was used for the management of the palatoradicular groove. Periodontal instrumentation, odontoradiculoplasty, placement of fissure sealant material and application of an enamel matrix derivative combined with bone grafting material were performed during regenerative therapy. Tooth 12 was restored with a full-coverage monolithic zirconia crown 6 months after regenerative therapy. This procedure had a successful outcome with a reduction in probing depth on the mid-palatal site of Tooth 12 to 3 mm, bone remodelling in the defect areas and minimum scarring at the 6-month review appointment. This case highlights the importance of an interdisciplinary approach in the management of periodontal destruction associated with palatoradicular grooves.

## 1. Introduction

Palatal groove, also known as palatogingival groove or palatoradicular groove, is a developmental, anomalous groove that can lead to the development of localised periodontal tissue breakdown or pulpal infections [1]. They are commonly found on the palatal aspect of the maxillary central and lateral incisors [2, 3] and appear as a funnel-like anomaly, predisposing the tooth to the accumulation of biofilm and calculus formation [2, 4–8]. Although genetic predisposition and local factors affecting tooth development have been researched, the aetiology of these root malformations is unknown [3]. Prevalence ranges from 1.9% to 18% and is seen more often in individuals of Asian descent [4, 5, 9–11].

Palatoradicular grooves begin at the junction of the cingulum with a lateral marginal ridge and extend apically [12]. Anomalies embryologically arise from a mild form of invagination from the folding of the enamel epithelium and are closely linked to dens in dente [9]. Invaginations can enter the pulp space as well as communicate with the periodontal ligament in an apical or lateral direction on the tooth root via a pseudoforamen [3, 12]. These grooves most commonly appear on the mid-palatal area (42.5%), followed by a distal aspect (30.1%) and then a mesial aspect (27.4%) [10]. Then, 58% of the grooves extend more than 5 mm from the CEJ, whereas 8.6% extend to the root apex [4]. Radiographically, they appear as radiolucent, parapulpal lines representing a radicular extension of the palatal groove [2, 13]. Localised periodontitis can be seen in the presence of palatoradicular grooves as these teeth have higher gingival index, plaque index, periodontal disease index scores and deeper probing depths when compared to teeth without grooves [5, 7, 8, 10, 13].

Palatoradicular grooves have been classified into three groups according to their severity [14]. Type I is when the groove is short, that is, it does not extend beyond the coronal third of the root. Type II is when the groove is long, that is, extending beyond the coronal third of the root but shallow. Type III is when the groove is long and deep, that is, extending beyond the coronal third of the root with involvement of the root canal system [14].

Nonsurgical periodontal therapy (NSPT) can be considered a blind procedure and direct visualisation of the root surfaces and residual deposits via periodontal surgery can improve the effectiveness of periodontal instrumentation. Direct access may be particularly beneficial if there is an intrabony defect, furcation involvement or a developmental groove such as the palatoradicular groove. Following periodontal destruction, there may be tissue contours that are difficult to maintain such as partial access to an intrabony defect, furcation area or a palatoradicular groove. Periodontal surgery can recontour the tissues for ideal plaque control by the patient as well as allowing the regeneration of tissue. Regenerative techniques can encourage new supporting structures to develop; however, the techniques are only suitable in specific circumstances such as within a contained defect which are two-or three-walled intrabony defects or Class II furcation defects [15].

## 2. Case Presentation

A 28-year-old female patient with a nonremarkable medical history presented to the periodontics department at the Oral Health Centre of Western Australia (OHCWA) in February 2024 with a presenting concern of a history of a periodontal abscess on Tooth 12. The patient was initially seen at her local government dental clinic regarding pain associated with Tooth 12. The general dentist at this clinic initiated endodontic treatment; however, the patient continued to have symptoms with the tooth despite multiple appointments for redressing the root canal system. The patient was then referred to the endodontics department of the OHCWA for further investigation. The palatoradicular groove was detected on Tooth 12, and the patient was referred to the periodontics department for management.

**Figure 1:**
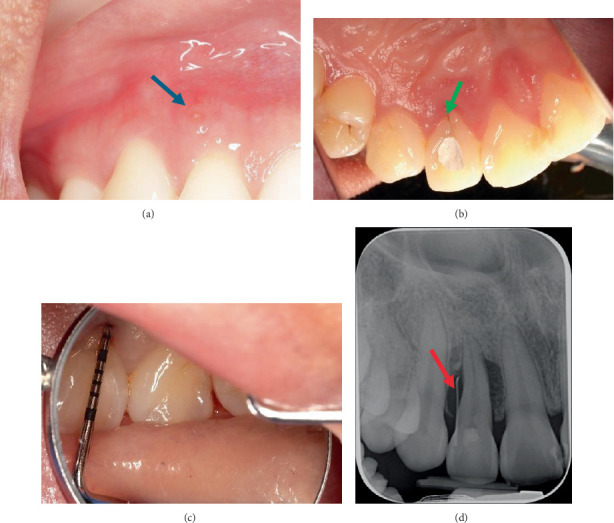
Preoperative photos of Tooth 12. (a) Buccal mucosa with draining sinus on distobuccal site (blue arrow). (b) Palatal aspect with palatal fissure (green arrow) and IRM restoration in access cavity. (c) Mid-palatal site with PPD of 7 mm and mild suppuration (purple arrow). (d) Periapical radiograph with GP point in draining sinus (red arrow).

During the initial presentation on 12 February 2024, it was noted that Tooth 12 had a draining sinus on the distobuccal mucosa (Figure 1a). A palatal fissure was observed in Tooth 12 (Figure 1b). The contralateral Tooth 22 also had a palatal fissure, but it was not as prominent as the palatal fissure on Tooth 12, as it was covered by a glass ionomer cement (GIC) restoration. Tooth 12 underwent an incomplete root canal treatment (RCT). The patient's periodontium had very little to no signs of plaque and calculus, with minimum bleeding on probing (BOP) as both her plaque index score, and her BOP score were under 10% using the Ainamo and Bay BOP score and the O'Leary et al. plaque score, respectively [16, 17]. No periodontal pockets were detected in her dentition, except for a 7 mm periodontal probing depth (PPD) with mild suppuration on the mid-palatal aspect of Tooth 12 (Figure 1c). No recession or mobility was detected in the tooth. The patient had a Class I edge-to-edge occlusion. Tooth 12 had no fremitus or interferences detected on excursive movements.

**Figure 2:**
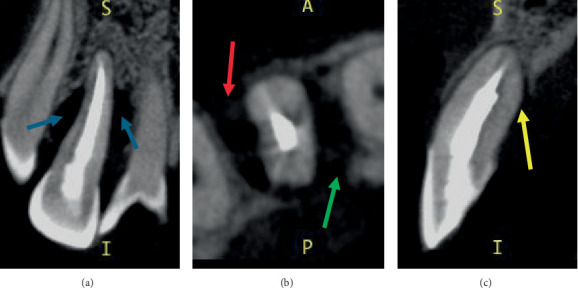
CBCT scan of Tooth 12. (a) Coronal section showing intrabony defects on mesial and distal aspects (blue arrows). (b) Transverse section showing the distal intrabony defect extending to the buccal aspect (red arrow) and the mesial intrabony defect extending to the palatal aspect (green arrow). (c) Sagittal section showing loss of bone on the palatal aspect (yellow arrow).

An intraoral periapical radiograph taken on 22 January 2024 (Figure 1d) with a gutta-percha (GP) point placed through the draining sinus showed angular, almost crater-like, alveolar bone loss on the mesial and distal aspects of Tooth 12 extending to the apical third of the root. There was no caries or periapical radiolucency present, and the tooth had evidence of intracanal medicament due to the incomplete RCT. The patient was referred for a cone beam computed tomography (CBCT) scan to assess the extent of the intrabony defect. The CBCT scan taken 2 months prior to surgery (Figures 2a, 2b and 2c) showed a two-wall intrabony defect on the mesial and distal aspects of Tooth 12, with the mesial aspect having a lack of alveolar bone on the palatal side whereas the distal aspect has a lack of alveolar bone on the buccal side.

The diagnosis for Tooth 12 was incomplete RCT with no signs of infection and clinically normal periapical tissues and a localised periodontal abscess due to a deep developmental palatal groove. The treatment plan for the management of Tooth 12 included NSPT, completion of endodontic treatment and periodontal regenerative surgery.

## 3. Procedure

**Figure 3:**
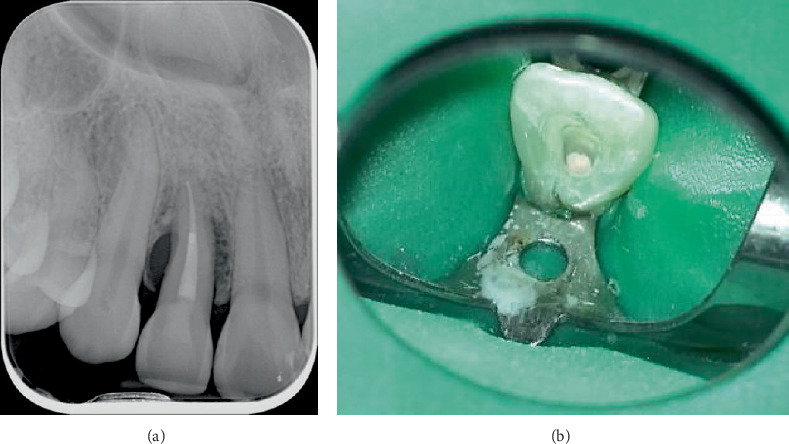
Obturation of Tooth 12. (a) Periapical radiograph. (b) Intraoral photograph.

Tooth 12 underwent NSPT first, on 21 March 2024, which involved instrumentation under local anaesthesia with an ultrasonic device (EMS Piezon Handpiece with PS tip) and hand instrument (Periodontal Hoe Scaler, Lateral), followed by application of 0.5% topical chlorhexidine gel (Curasept ADS 350) in the periodontal pocket on the mid-palatal site via a syringe with a fine tip needle to reach the depth of the periodontal pocket. The patient was given specific oral hygiene instructions for the management of Tooth 12 which involved application of the 0.5% topical chlorhexidine gel with an end-tufted toothbrush (TePe) along the palatal aspect of the tooth. The obturation of the canal in Tooth 12 (Figure 3a,b) was completed on 1 May 2024, 1 week prior to the periodontal surgery on 8 May 2024. The palatoradicular groove did not extend into the pulpal chamber.

**Figure 4:**
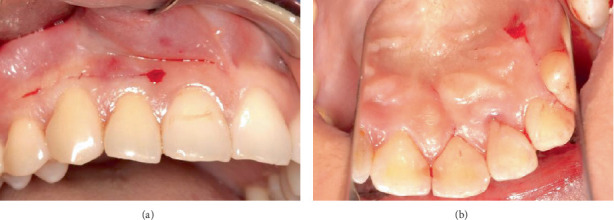
Incisions. (a) Submarginal horizontal incision on labial mucosa. (b) Intrasulcular and vertical releasing incisions on palatal mucosa.

Surgical intervention for the management of the intrabony defects and palatoradicular groove on Tooth 12 involved access flap surgery, regenerative therapy, odontoplasty and radiculoplasty. The procedure was performed under local anaesthesia. A minimal access papilla-sparing regenerative technique (MAPSRT), a novel, site-specific modification not previously reported, was used on the labial aspect. This involved a submarginal horizontal incision (Figure 4a) being made using a No. 15 scalpel blade on the labial mucosa approximately 2 mm apical from the gingival margin to preserve the interdental papillae, extending from the distal of Tooth 13 to the distal of Tooth 11. A trapezoidal flap was used on the palatal aspect. Intrasulcular incisions were placed using a No. 15c scalpel blade on the palatal aspect from the distal of Tooth 13 to the mesial of Tooth 11, followed by the placement of vertical releasing incisions using a No. 15 scalpel blade on the distal of Tooth 13 and the mesial of Tooth 11 (Figure 4b).

**Figure 5:**
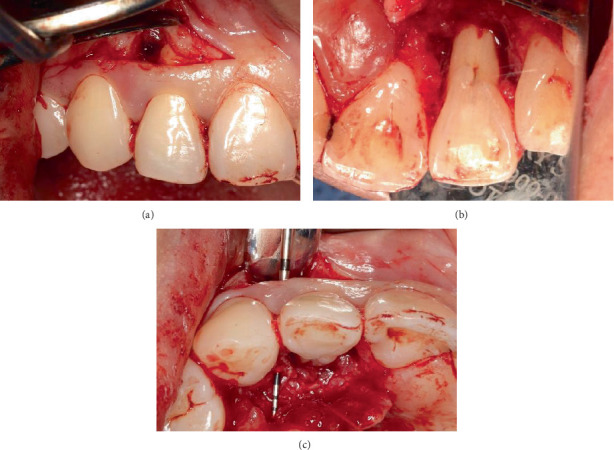
Elevation of mucoperiosteal flap. (a) Buccal aspect. (b) Palatal aspect. (c) Extent of infrabony defect on distobuccal aspect.

A full mucoperiosteal flap was raised using Buser and Molt periosteal elevators on the buccal aspect, revealing the intrabony defect on the distobuccal aspect of Tooth 12 (Figure 5a) and on the palatal aspect (Figure 5b), revealing the palatoradicular groove and the intrabony defect on the mesial and distal sites. The palatoradicular groove appeared to be Type II as it extended beyond the coronal third of the root. The interdental papillae on the buccal aspect of Teeth 13, 12 and 11 were left intact. After raising both the buccal and palatal mucoperiosteal flaps, it was evident that the intrabony defect on the distal aspect of Tooth 12 extended from the buccal to the palatal aspects, as shown by the penetration of the UNC 15 periodontal probe (Figure 5c).

**Figure 6:**
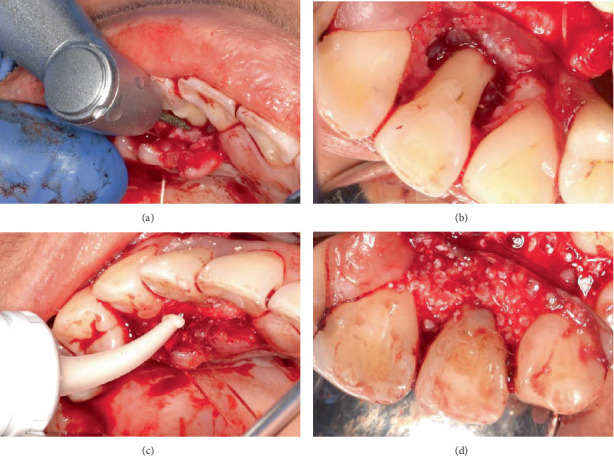
Management of palatoradicular groove on Tooth 12. (a) Smoothing of palatoradicular groove. (b) Result after odontoradiculoplasty. (c) Application of GIC restoration on palatal fissure. (d) Coverage of palatal fissure with GIC restoration.

Interdental tissues were removed with the No. 15c scalpel blade as well as with the Younger Good Curette. Mechanical instrumentation of Tooth 12 was performed using hand instruments, Gracey's 3/4 and Columbia 4L/4R. The palatoradicular groove on the root of Tooth 12 was smoothed using fine diamond burs from the Komet bur kit with a red-band surgical handpiece (Figure 6a,b). A GIC restoration was placed only on the palatal fissure on the cingulum of Tooth 12 (Figure 6c,d).

**Figure 7:**
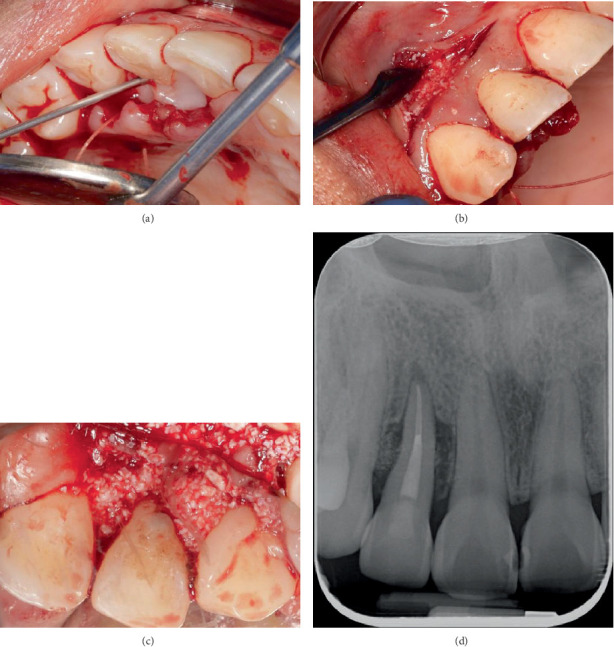
(a) Application of Straumann PrefGel in intrabony defect. Application of xenograft with EMD in (b) buccal aspect and (c) palatal aspect. (d) Periapical radiograph with Bio-Oss/EMD filling intrabony defect.

Regenerative therapy was performed on Tooth 12. This involves the use of a biologically active agent, an enamel matrix derivative (EMD) commercially known as Emdogain (Straumann), in combination with a bone graft to act as a scaffold owing to the viscous nature of the EMD [18]. In this case, the deproteinised bovine bone mineral commercially available as Bio-Oss (Geistlich) was used as the xenograft. The smear layer was removed with EDTA-containing gel, Straumann PrefGel, on the buccal and palatal aspects of the intrabony defect sites and left for 2 min (Figure 7a). The PrefGel was then rinsed with sterile saline, and the area was dried with sterile gauze. Straumann Emdogain was placed on the most apical part of the exposed root surface of Tooth 12. Subsequently, Emdogain was mixed with Geistlich Bio-Oss granules and placed in increments to fill the intrabony defects on the buccal and palatal aspects (Figure 7b,c). An intraoral periapical radiograph was obtained to ensure that the infrabony defects were sufficiently filled with the xenograft mixed with the EMD (Figure 7d).

**Figure 8:**
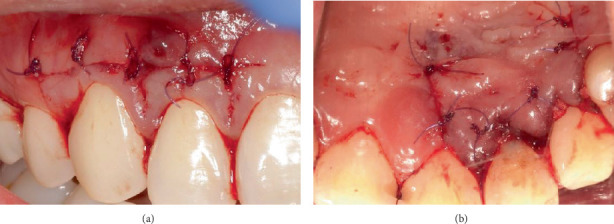
Flap closure on (a) buccal and (b) palatal aspects.

Flap closure for the surgical site of Tooth 12 was achieved via five simple interrupted sutures on the buccal aspect from Tooth 13 distal to Tooth 11 distal, two vertical mattress sutures on the interdental papillae of Teeth 13/12 and Teeth 12/11 sites and four simple interrupted sutures for the vertical releasing incisions on the palatal aspects (Figure 8a,b). Glycolon sutures (6.0) with 13 mm reverse cutting needles were used for flap closure. Haemostasis was achieved, and postoperative management was provided to the patient, which included the administration of 500 mg of paracetamol as an analgesic, 0.12% chlorhexidine mouthrinse (Curasept ADS 212) two times a day for 2 weeks and the use of a surgical toothbrush (Curaprox) to clean the surgical site, commencing 2 weeks after the procedure.

## 4. Postoperative Management

**Figure 9:**
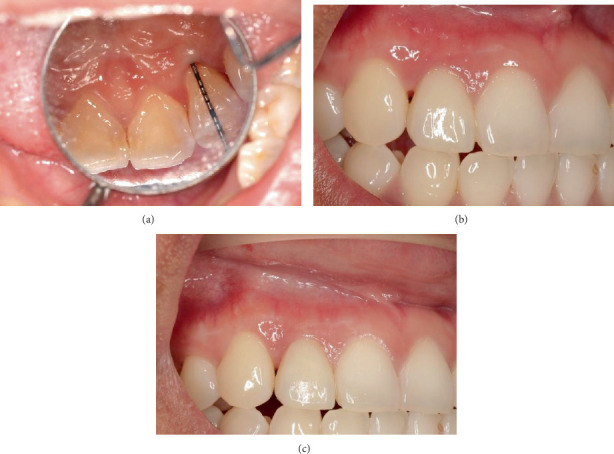
(a) Palatal aspect of Tooth 12 at 6-month review with PPD of 3 mm on mid-palatal aspect. Labial aspect of Tooth 12 at (b) 6-month and (c) 1-year review.

**Figure 10:**
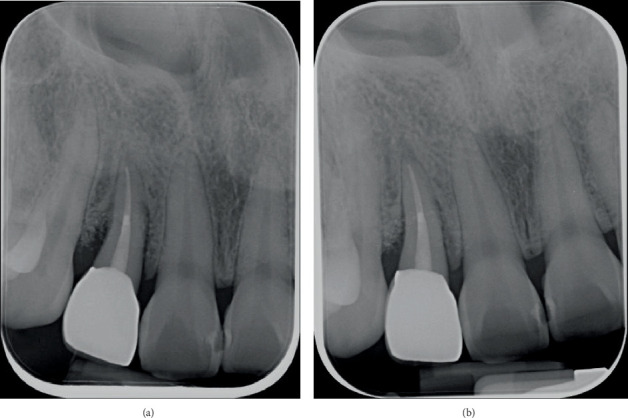
Periapical radiograph of Tooth 12 at (a) 6-month and (b) 1-year review showing bone remodelling in areas of intrabony defect.

The patient had a review appointment 2 weeks after surgery, which involved suture removal, and then had another review appointment at 3 months. Evidence of soft tissue healing without any adverse events was observed. There were no signs of erythema or oedema, and minimal scarring was evident along the incision line. At the 6-month review, the probing depth on the mid-palatal site of Tooth 12 was reduced from 7 to 3 mm (Figure 9a). As the tooth had undergone endodontic treatment and an edge-to-edge occlusion, a full coverage monolithic zirconia crown was fabricated by a general dentist at OHCWA (Figure 9b), followed by a review appointment 1 year after the regenerative therapy (Figure 9c). The periapical radiographs taken at the 6-month and 1-year review appointments showed bone remodelling in the area where the intrabony defect was (Figure 10a,b). The patient was very satisfied with the results of the multidisciplinary approach for the management of the palatoradicular groove.

## 5. Discussion

The CBCT was useful in determining the extent of the intrabony defect on Tooth 12, as it allowed for visualisation of the anatomic configuration in all dimensions [19]. A small field of view CBCT was obtained of Tooth 12 to follow the ALARA principle of as low radiation as possible [20]. However, it was difficult to visualise the extent of the palatoradicular groove radiographically, and the only clinical manifestations were the increased probing depth of 7 mm on the mid-palatal site of Tooth 12 with suppuration. As endodontic treatment had already commenced prior to the patient being referred to OHCWA, it was difficult to determine whether the tooth was vital prior to the endodontic treatment. A clinical photograph of the access cavity of Tooth 12 taken after obturation showed that the palatoradicular groove did not extend into the pulp chamber, suggesting that this tooth may not have required endodontic treatment. However, if the groove or invagination extends into the pulpal space, thus creating a pulpal condition, endodontic treatment is indicated [21]. On the other hand, the study by Jepsen et al. stated that “prophylactic” endodontic treatment is recommended just prior to or after regenerative therapy on teeth with severe periodontal destruction extending to the apical third of the tooth to avoid the undesired outcome of an advanced endo–perio lesion [22]. Therefore, the completion of the endodontic treatment on Tooth 12 1 week prior to the regenerative therapy would have been done regardless of the extent of the invagination as there was evidence of advanced periodontal destruction.

Flap design to allow access to the defect site for periodontal instrumentation and the application of regenerative materials is crucial. Many flap designs were considered for the management of the palatoradicular groove on Tooth 12: Kirkland flap, modified papilla preservation technique (MPPT) by Cortellini et al., simplified papilla preservation flap (SPPT) by Cortellini and Tonetti, minimally invasive surgical technique (MIST) by Cortellini and Tonetti and modified minimally invasive surgical technique (M-MIST) by Cortellini and Tonetti [23–27]. These various flap designs are usually applied in cases where residual deep periodontal pockets are still present after the completion of NSPT. These sites are commonly located interproximally. In contrast, the bony defect of the palatoradicular groove is on the palatal aspect. Therefore, on the buccal aspect, we utilised what we have termed the MAPSRT, a novel, site-specific modification not previously reported. This horizontal incision 2 mm apical to the gingival margin allowed defect access while preserving the buccal papillae and avoiding vertical releases, thereby maintaining vascular integrity in the aesthetic zone. Periodontal instrumentation and bone graft placement were performed through this minimal access, in line with contemporary minimally invasive regenerative approaches. On the palatal aspect, however, a trapezoidal flap with vertical releases was necessary to ensure optimal visibility and access to the intrabony defect and palatoradicular groove, features located in a nonaesthetic area where aesthetic constraints were less critical. Midline interproximal incisions were employed to preserve the supraperiosteal blood supply [28, 29]. The use of the buccal MAPSRT eliminated the need for papilla preservation on the palatal side, allowing us to balance minimally invasive principles on the buccal aspect with the surgical access requirements dictated by the complex anatomy on the palatal aspect. This dual-flap approach was therefore chosen to maximise regenerative outcomes while respecting both anatomical and aesthetic constraints.

Management of the palatoradicular groove can involve a simpler approach such as cleaning and sealing the defect as outlined by Pitt Ford or via regenerative therapy as demonstrated by Anderegg and Metzler [30, 31]. This involved surgery via removal of granulation tissue, instrumentation of the site and placement of a polytetrafluoroethylene membrane covering the groove.

Given that the groove includes both equigingival and supragingival components, there is concern about the potential wash-out of hydraulic calcium silicate cements (HCSCs) in these areas due to increased exposure to gingival and oral fluids, particularly if the material has not yet fully set. While Biodentine, proposed as an alternative, does offer a faster setting time compared to other HCSCs like MTA, which is advantageous during surgical procedures, recent studies by Falkowska et al. and Falkowska-Ostrowska and Dura have demonstrated its poor washout resistance when used as a root-end filling material in endodontic surgery [32, 33]. If Biodentine shows limited resistance even when used as a thick root-end plug, its use in smaller volumes, such as for sealing a groove, raises greater concern regarding its stability and retention in situ. Also, there are no specific studies on any materials that could be used to restore a palatoradicular groove.

In contrast, GIC chemically bonds to both enamel and dentine without the need for a bonding agent, creating a strong, stable bond reducing the risk of microleakage and enhancing seal integrity [34, 35]. In addition to this, GIC releases fluoride over time, which can prevent bacterial growth and remineralise the tooth structure [34, 35]. The dimensional stability of GIC is better compared to Biodentine due to its minimum expansion and contraction during setting, resulting in a long-lasting seal, whereas Biodentine may have slight dimensional changes during maturation that can influence marginal integrity [36]. Also, GIC is more tolerant in moist environments and has a lower solubility, making it resistant to oral fluids and maintaining its seal over time [34, 35]. Therefore, in this case, GIC was selected as a more suitable material due to its fast-setting properties and comparatively better resistance to wash-out.

Regenerative periodontal therapy involves a variety of surgical procedures that aim at achieving healing of periodontal destruction by developing new periodontium, instead of repairing with a long junctional epithelial attachment. These techniques include guided tissue regeneration, EMD and growth factors. EMD (known commercially as Straumann Emdogain) consists of enamel matrix proteins and has been found to encourage cementum formation and periodontal regeneration [37]. Evidence of periodontal regeneration and clinical improvements after using EMD in intrabony defects is present in histological and clinical studies, but concerns have been raised regarding the viscous character of EMD which may not be sufficient to prevent flap collapse in periodontal defects with complex anatomy [18]. This can result in limitation of the available space for regeneration preventing the ideal clinical outcomes from being obtained. Hence, to prevent this complication and improve the clinical results, various combinations of EMD with different types of grafting materials have been used [18]. For the regenerative therapy, as part of the management of the palatoradicular groove on Tooth 12, Straumann Emdogain was used in combination with Bio-Oss (Geistlich Pharma AG), a well-documented xenograft consisting of a highly purified anorganic bone matrix mineral of bovine origin, which acted as a scaffold for bone deposition [18]. No barrier membrane was used as the intrabony defect surrounding Tooth 12 was a contained defect.

## 6. Conclusion

A multidisciplinary approach to the management of a palatoradicular groove on a maxillary lateral incisor has shown to be successful. This involved the combination of endodontic treatment, odontoradiculoplasty, application of fissure sealant, regenerative therapy and fabrication of a full coverage crown. The conservative nature of the flap design on the buccal aspect has allowed for the interdental papillae to be intact, whereas having full access on the palatal aspect via the conventional Kirkland flap has allowed for the adaptation of the regenerative material into the intrabony defect, leading to improvements in the PPD on the mid-palatal aspect of Tooth 12 from 7 to 3 mm over a 6-month period.

## Data Availability

The data that support the findings of this study are available from the corresponding author upon reasonable request.
